# Supplementary material for: Clinical streptococcal isolates, distinct from Streptococcus pneumoniae, but containing the β-glucosyltransferase tts gene and expressing serotype 37 capsular polysaccharide
Source: PeerJ. 2017 Jul 18;5:e3571. doi: 10.7717/peerj.3571 (PMC5518733; doi:10.7717/peerj.3571)
Supplement: Table S1 [file peerj-05-3571-s002.docx]

**Supplementary Table 1.** ENA accession numbers for genomic data from isolates used in this study.

| SAMPLE | ENA Project | Accession |
| --- | --- | --- |
| PHESPD0338 | PRJEB14267 | ERS1193636 |
| PHESPD0344 | PRJEB14267 | ERS1193642 |
| PHESPD0356 | PRJEB14267 | ERS1193654 |
| PHESPD0383 | PRJEB14267 | ERS1193681 |
| PHESPV0691 | PRJEB14267 | ERS1194859 |
| PHESPV1034 | PRJEB14267 | ERS1195202 |
| PHESPV1405 | PRJEB14267 | ERS1195570 |
| PHESPD0363 | PRJEB14267 | ERS1193661 |
| PHESPV0789 | PRJEB14267 | ERS1194957 |
| PHESPV1119 | PRJEB14267 | ERS1195287 |
| PHESPD0355 | PRJEB14267 | ERS1193653 |
| SSI-37 | PRJEB14267 | ERS1193365 |
| PHESPD0357 | PRJEB14267 | ERS1193655 |
| PHENP00001 | PRJEB20507 | ERS1674955 |
| PHENP00012 | PRJEB20507 | ERS1674956 |
| PHENP00011 | PRJEB20507 | ERS1674957 |
| PHENP00002 | PRJEB20507 | ERS1674958 |
| PHENP00016 | PRJEB20507 | ERS1674959 |
| PHENP00010 | PRJEB20507 | ERS1674960 |
| PHENP00009 | PRJEB20507 | ERS1674961 |
| PHENP00018 | PRJEB20507 | ERS1674962 |
| PHENP00017 | PRJEB20507 | ERS1674963 |
| PHENP00003 | PRJEB20507 | ERS1674964 |
| PHENP00008 | PRJEB20507 | ERS1674965 |
| PHENP00005 | PRJEB20507 | ERS1674966 |
| PHENP00006 | PRJEB20507 | ERS1674967 |
| PHENP00007 | PRJEB20507 | ERS1674968 |
| PHENP00014 | PRJEB20507 | ERS1674969 |
| PHENP00013 | PRJEB20507 | ERS1674970 |
| PHENP00015 | PRJEB20507 | ERS1674971 |
| BAA-960 | PRJEB20507 | ERS1674972 |
