# Supplementary material for: Clinical streptococcal isolates, distinct from Streptococcus pneumoniae, but containing the β-glucosyltransferase tts gene and expressing serotype 37 capsular polysaccharide
Source: PeerJ. 2017 Jul 18;5:e3571. doi: 10.7717/peerj.3571 (PMC5518733; doi:10.7717/peerj.3571)
Supplement: Table S2 [file peerj-05-3571-s003.docx]

Supplementary Table 2. Streptococcal species used in the extended kmerID database

| Genus | Species | Number of reference genomes |
| --- | --- | --- |
| *Streptococcus* | *agalactiae* | 260 |
| *Streptococcus* | *anginosus* | 8 |
| *Streptococcus* | *australis* | 2 |
| *Streptococcus* | *bovis* | 1 |
| *Streptococcus* | *caballi* | 1 |
| *Streptococcus* | *canis* | 1 |
| *Streptococcus* | *constellatus* | 6 |
| *Streptococcus* | *criceti* | 1 |
| *Streptococcus* | *cristatus* | 2 |
| *Streptococcus* | *didelphis* | 1 |
| *Streptococcus* | *downei* | 1 |
| *Streptococcus* | *dysgalactiae* | 7 |
| *Streptococcus* | *entericus* | 1 |
| *Streptococcus* | *equi* | 5 |
| *Streptococcus* | *equinus* | 1 |
| *Streptococcus* | *ferus* | 1 |
| *Streptococcus* | *gallolyticus* | 4 |
| *Streptococcus* | *gordonii* | 1 |
| *Streptococcus* | *henryi* | 1 |
| *Streptococcus* | *ictaluri* | 1 |
| *Streptococcus* | *infantarius* | 2 |
| *Streptococcus* | *infantis* | 6 |
| *Streptococcus* | *iniae* | 4 |
| *Streptococcus* | *intermedius* | 6 |
| *Streptococcus* | *lutetiensis* | 1 |
| *Streptococcus* | *macacae* | 1 |
| *Streptococcus* | *macedonicus* | 1 |
| *Streptococcus* | *marimammalium* | 1 |
| *Streptococcus* | *massiliensis* | 2 |
| *Streptococcus* | *merionis* | 1 |
| *Streptococcus* | *minor* | 1 |
| *Streptococcus* | *mitis* | 15 |
| *Streptococcus* | *mutans* | 68 |
| *Streptococcus* | *oligofermentans* | 1 |
| *Streptococcus* | *oralis* | 10 |
| *Streptococcus* | *orisratti* | 1 |
| *Streptococcus* | *ovis* | 1 |
| *Streptococcus* | *parasanguinis* | 6 |
| *Streptococcus* | *parauberis* | 5 |
| *Streptococcus* | *pasteurianus* | 1 |
| *Streptococcus* | *peroris* | 1 |
| *Streptococcus* | *pneumoniae* | 257 |
| *Streptococcus* | *porcinus* | 1 |
| *Streptococcus* | *pseudopneumoniae* | 3 |
| *Streptococcus* | *pseudoporcinus* | 2 |
| *Streptococcus* | *pyogenes* | 23 |
| *Streptococcus* | *ratti* | 2 |
| *Streptococcus* | *salivarius* | 7 |
| *Streptococcus* | *sanguinis* | 22 |
| *Streptococcus* | *sobrinus* | 1 |
| *Streptococcus* | *spp.* | 3 |
| *Streptococcus* | *suis* | 19 |
| *Streptococcus* | *thermophilus* | 9 |
| *Streptococcus* | *thoraltensis* | 1 |
| *Streptococcus* | *tigurinus* | 2 |
| *Streptococcus* | *uberis* | 1 |
| *Streptococcus* | *urinalis* | 2 |
| *Streptococcus* | *vestibularis* | 2 |
|  | Total references | 798 |
|  | Total species | 58 |
