# Supplementary material for: Clinical streptococcal isolates, distinct from Streptococcus pneumoniae, but containing the β-glucosyltransferase tts gene and expressing serotype 37 capsular polysaccharide
Source: PeerJ. 2017 Jul 18;5:e3571. doi: 10.7717/peerj.3571 (PMC5518733; doi:10.7717/peerj.3571)
Supplement: Table S3 — *Top hit was always serotype 37 tts gene except for BAA-960T which had 0% coverage for tts, therefore the serotype in the table is the top hit serotype. [file peerj-05-3571-s004.docx]

Supplementary Table 3. Coverage on mapping to the capsular operon reference sequence for the next hit after serotype 37 *tts* during *Streptococcus pneumoniae* serotyping workflow.

| Isolate number | Species | Second hit serotype reference | % coverage |
| --- | --- | --- | --- |
| PHESPV0789 | *Streptococcus pneumoniae* | 33A | 98.3 |
| PHESPV1119 | *Streptococcus pneumoniae* | 33A | 98.3 |
| PHESPD0344 | *Streptococcus pneumoniae* | 33A | 98.1 |
| PHESPV0691 | *Streptococcus pneumoniae* | 33A | 98.0 |
| PHESPD0383 | *Streptococcus pneumoniae* | 33A | 98.0 |
| PHESPD0356 | *Streptococcus pneumoniae* | 33A | 97.9 |
| PHESPD0363 | *Streptococcus pneumoniae* | 33A | 97.8 |
| SSI-37 | *Streptococcus pneumoniae* | 33A | 97.7 |
| PHESPD0355 | *Streptococcus pneumoniae* | 33A | 97.7 |
| PHESPD0338 | *Streptococcus pneumoniae* | 33A | 97.1 |
| PHESPV1405 | *Streptococcus pneumoniae* | 33F | 96.9 |
| PHESPV1034 | *Streptococcus pneumoniae* | 33F | 96.4 |
| PHENP00005 | *Streptococcu*s sp. | 36 | 19.6 |
| PHESPD0357 | *Streptococcus* sp. | 25A/25F | 13.7 |
| PHENP00003 | *Streptococcus* sp. | 25A | 9.3 |
| PHENP00006 | *Streptococcus* sp. | 36 | 9.2 |
| PHENP00007 | *Streptococcus* sp. | 25A/25F | 8.7 |
| PHENP00002 | *Streptococcus* sp. | 1 | 8.6 |
| PHENP00001 | *Streptococcus* sp. | 1 | 8.6 |
| BAA-960^T^ | S*treptococcus pseudopneumoniae* | 36* | 26.4 |

*Top hit was always serotype 37 *tts* gene except for BAA-960^T^ which had 0% coverage for *tts*, therefore the serotype in the table is the top hit serotype.
