# Supplementary material for: Clinical streptococcal isolates, distinct from Streptococcus pneumoniae, but containing the β-glucosyltransferase tts gene and expressing serotype 37 capsular polysaccharide
Source: PeerJ. 2017 Jul 18;5:e3571. doi: 10.7717/peerj.3571 (PMC5518733; doi:10.7717/peerj.3571)
Supplement: Table S4 — * additional amino acids seen in PHENP00001 and PHENP00002. §substitution only seen in SSI-37 reference strain. ¥ a value of 1.000 is very hydrophobic, a value of 0.000 is very hydrophilic. [file peerj-05-3571-s005.docx]

Supplementary Table 4. Details of amino acid properties for substitution positions with Negative BLOSUM62 scores compared to reference sequence amino acid (aa).

| **aa position** | **tts reference aa** | **reference aa property** | **Reference aa Hydrophobicity¥** | **Substituted position aa** | **Substitution property** | **Substitution Hydrophobicity** |
| --- | --- | --- | --- | --- | --- | --- |
| 85* | - | - | - | Isoleucine | Non-polar, no H-bonds | 1.000 |
| 86* | - | - | - | Tyrosine | Polar, 3 potential H-bonds, aromatic | 0.714 |
| 99 | Alanine | Neutral, no H-bonds | 0.806 | Asparagine | Acidic, negative charge, 4 potential H-bonds | 0.417 |
| 247 | Tyrosine | Polar, 3 potential H-bonds | 0.714 | Arginine | Basic, positive charge, 7 potential H-bonds | 0.000 |
| 341 § | Tryptophan | Non-Polar, No H-bond, aromatic | 0.854 | Glycine | Non-polar, no H-bonds | 0.770 |
| 389 | Valine | Non-Polar, No H-bonds | 0.923 | Phenylalanine | Non-polar, no H-bonds, aromatic | 0.951 |
| 473 | Threonine | Polar, 3 potential H-bonds | 0.634 | Isoleucine | Non-polar, no H-bonds | 1.000 |
| 476 | Threonine | Polar, 3 potential H-bonds | 0.634 | Methionine | Non-polar, no H-bonds | 0.811 |

* additional amino acids seen in PHENP00001 and PHENP00002.

§ substitution only seen in SSI-37 reference strain.

¥ a value of 1.000 is very hydrophobic, a value of 0.000 is very hydrophilic.
