# Supplementary material for: Clinical streptococcal isolates, distinct from Streptococcus pneumoniae, but containing the β-glucosyltransferase tts gene and expressing serotype 37 capsular polysaccharide
Source: PeerJ. 2017 Jul 18;5:e3571. doi: 10.7717/peerj.3571 (PMC5518733; doi:10.7717/peerj.3571)
Supplement: Table S5 — The maximum and minimum JI values denote a range in which the MCC is 95% of the maximum or above. [file peerj-05-3571-s006.docx]

Supplementary Table 5. Maximum Matthew’s correlation coefficient (MCC) for 18 streptococcal species when separating them by pairwise kmer Jaccard index of set similarity (JI) comparison from other streptococcal species. The maximum and minimum JI values denote a range in which the MCC is 95% of the maximum or above.

| **Streptococcal species** | **Max MCC** | **MCC limit** | **Min JI** | **Max JI** |
| --- | --- | --- | --- | --- |
| *S. sanguinis* | 0.94 | 0.89 | 0.06 | 0.2 |
| *S. mutans* | 1.00 | 0.95 | 0.009 | 0.6 |
| *S. pneumoniae* | 0.99 | 0.94 | 0.06 | 0.6 |
| *S. anginosus* | 1.00 | 0.95 | 0.15 | 0.2 |
| *S. infantis* | 0.85 | 0.80 | 0.04 | 0.08 |
| *S. thermophilus* | 1.00 | 0.95 | 0.2 | 0.5 |
| *S. dysgalactiae* | 1.00 | 0.95 | 0.15 | 0.3 |
| *S. equi* | 1.00 | 0.95 | 0.02 | 0.4 |
| *S. suis* | 1.00 | 0.95 | 0.02 | 0.25 |
| *S. parauberis* | 1.00 | 0.95 | 0.02 | 0.6 |
| *S. oralis* | 0.84 | 0.79 | 0.15 | 0.15 |
| *S. mitis* | 0.33 | 0.28 | 0.2 | 0.2 |
| *S. constellatus* | 0.89 | 0.84 | 0.2 | 0.4 |
| *S. salivarius* | 0.85 | 0.80 | 0.09 | 0.25 |
| *S. agalactiae* | 0.99 | 0.94 | 0.02 | 0.55 |
| *S. parasanguinis* | 1.00 | 0.95 | 0.06 | 0.2 |
| *S. pyogenes* | 1.00 | 0.95 | 0.15 | 0.6 |
| *S. intermedius* | 0.80 | 0.75 | 0.45 | 0.5 |
