# Supplementary material for: Clinical streptococcal isolates, distinct from Streptococcus pneumoniae, but containing the β-glucosyltransferase tts gene and expressing serotype 37 capsular polysaccharide
Source: PeerJ. 2017 Jul 18;5:e3571. doi: 10.7717/peerj.3571 (PMC5518733; doi:10.7717/peerj.3571)
Supplement: Table S6 [file peerj-05-3571-s007.docx]

Supplementary Table 6. Nucleotide differences within and between the groups of organisms used for the whole genome SNP analysis.

| Organism group | Between group differences | | | Within group differences |
| --- | --- | --- | --- | --- |
|  | Number of nucleotides | | | |
| Non-pneumococcal *tts* positive |  |  |  | 5327.3 |
| *Streptococcus pneumoniae* | 6359.3 |  |  | 1545.2 |
| *Streptococcus mitis* | 7661.514 | 6853.7 |  | 7811.4 |
| *Streptococcus pseudopneumoniae* | 7123.5 | 5857.8 | 7870.1 | 3555.7 |
