# Supplementary material for: Clinical streptococcal isolates, distinct from Streptococcus pneumoniae, but containing the β-glucosyltransferase tts gene and expressing serotype 37 capsular polysaccharide
Source: PeerJ. 2017 Jul 18;5:e3571. doi: 10.7717/peerj.3571 (PMC5518733; doi:10.7717/peerj.3571)
Supplement: Table S7 — COPD, chronic obstructive pulmonary disease. [file peerj-05-3571-s008.docx]

Supplementary Table 7. -Clinical details for non-pneumococcal *tts* positive isolates

| **Isolate number** | **Isolation site** | **Presentation** | **Comorbidities** | **Died** |
| --- | --- | --- | --- | --- |
| PHESPD0357 | Blood | Pneumonia | Congenital Mitochondrial Cytopathy | Y |
| PHENP00003 | Blood | Pneumonia | Not known |  |
| PHENP00005 | Sputum | ?Cough | Not known |  |
| PHENP00006 | Sputum | Pneumonia | Not known |  |
| PHENP00001 | Sputum | Pneumonia | End Stage COPD | Y |
| PHENP00002 | Sputum | Not known | Liver Cirrhosis | Y |
| PHENP00007 | Blood | Bacteraemia | ?Immunosuppression |  |

COPD, chronic obstructive pulmonary disease
